# Pea Albumin Alleviates Oleic Acid-Induced Lipid Accumulation in LO2 Cells Through Modulating Lipid Metabolism and Fatty Acid Oxidation Pathways

**DOI:** 10.3390/foods13213482

**Published:** 2024-10-30

**Authors:** Bing Fang, Jie Luo, Zhengwu Cui, Rong Liu, Pengjie Wang, Jian Zhang

**Affiliations:** 1 Department of Nutrition and Health, China Agricultural University, Beijing 100193, China; bingfang@cau.edu.cn (B.F.); 16653221635@163.com (Z.C.); liurong@cau.edu.cn (R.L.); wpj1019@cau.edu.cn (P.W.); 2 Food Laboratory of Zhongyuan, Luohe 462300, China; 3 College of Food Science and Technology, Hunan Agricultural University, Changsha 410114, China; luojie@hunau.edu.cn

**Keywords:** pea albumin, LO2 cell, lipid accumulation, oleic acid

## Abstract

Excessive lipid accumulation in the liver can cause NAFLD, leading to chronic liver injury. To relieve liver lipid accumulation by dietary proteins, this study used oleic acid (OA) induction to establish a stable in vitro LO2 cell lipid accumulation model. This model was used to explore the mechanism by which pea albumin (PA) regulates lipid levels in LO2 cells. PA has been shown to ameliorate OA-induced lipid accumulation in LO2 cells by reducing the aggregation of intracellular lipid droplets and lowering cell TG and TC levels. In addition, it can alleviate OA-induced LO2 cell damage and oxidative stress, reduce cellular ALT and AST secretion, lower cellular MDA levels, and increase GSH-Px viability. Regulation of lipid metabolism in LO2 cells involves inhibiting the cellular lipid synthesis pathway and activating the expression of proteins related to the triglyceride catabolic and fatty acid oxidation pathways. PA contributes to regulating lipid accumulation in LO2 cells. This study provides new insights into alleviating liver fat accumulation and a theoretical basis for exploring the mechanism of protein regulation of liver cell lipid metabolism.

## 1. Introduction

Non-alcoholic fatty liver disease (NAFLD), recently redefined as metabolic (dysfunction)-associated fatty liver disease (MAFLD), is the most prevalent chronic liver disease globally, with its incidence rising sharply, particularly in developed countries and emerging economies [1]. NAFLD affects approximately 25% of the global population and is characterized by excessive hepatic fat accumulation in individuals with minimal or no alcohol intake [2,3]. This condition includes a broad range of liver disorders, from simple hepatic steatosis, characterized by fat accumulation without inflammation, to the more severe non-alcoholic steatohepatitis (NASH), which involves inflammation and varying levels of fibrosis [4]. In its most advanced stages, NAFLD can progress to cirrhosis and hepatocellular carcinoma (HCC) [5]. The rising global incidence of NAFLD poses substantial challenges to public health systems and threatens individual well-being. The pathogenesis of NAFLD is closely linked to impaired hepatic lipid metabolism, leading to abnormal accumulation of triglycerides (TGs) in hepatocytes [6,7]. This initial lipid accumulation is a key factor in liver dysfunction and contributes to the progression of NAFLD to more advanced stages. NAFLD development is primarily attributed to an imbalance between lipid acquisition and clearance, characterized by increased dietary fat intake, uptake of circulating free fatty acids, and de novo lipogenesis, alongside reduced lipid disposal through fatty acid oxidation and export via very low-density lipoproteins (VLDLs) [8,9]. Under normal physiological conditions, the liver maintains lipid homeostasis through the coordinated regulation of these processes. However, in NAFLD, this balance is disrupted, with enhanced lipogenesis and diminished fatty acid oxidation due to insulin resistance and mitochondrial dysfunction [10]. Additionally, impaired VLDL secretion further exacerbates lipid retention in hepatocytes [11]. The resulting lipid accumulation not only triggers oxidative stress and liver cell damage but also promotes the progression of more severe conditions, including fibrosis and NASH [12]. Therefore, key strategies for alleviating NAFLD and preventing severe liver diseases include inhibiting hepatic de novo lipogenesis, enhancing lipid breakdown, and promoting fatty acid oxidative metabolism.

Current strategies to prevent and treat hepatic lipid accumulation in NAFLD primarily focus on lifestyle modifications, pharmacological interventions, and, in severe cases, surgery. While diet and exercise are fundamental to NAFLD management, their long-term adherence is often challenging. Pharmacological agents targeting lipid metabolism pathways, such as PPAR agonists and SREBP inhibitors, have shown potential in preclinical studies, but no specific drug has been approved for NAFLD treatment due to concerns over safety, efficacy, and prolonged administration [13,14,15]. Bariatric surgery is considered for patients with obesity-related NAFLD, though its invasive nature limits its applicability. These limitations emphasize the need for alternative, more accessible therapeutic strategies to manage hepatic lipid accumulation and prevent disease progression. Plant proteins have attracted considerable interest for their role in managing metabolic disorders, particularly NAFLD. Research has shown that plant proteins, with their distinctive bioactive peptides and amino acid compositions, can modulate lipid metabolism by influencing key processes such as lipogenesis, fatty acid oxidation, and lipid transport. Studies on phycobiliprotein peptide extracts from *Arthrospira platensis* further demonstrate their ability to regulate hepatic lipid metabolism by influencing the dynamics of neutral lipids and phospholipids [16]. Soy glycinin-derived octapeptide has been demonstrated to reduce lipid accumulation in HepG2 cells by activating the SIRT1/AMPK pathway [17]. Plant-based proteins are also associated with reduced inflammation and oxidative stress, both of which are critical factors in the pathogenesis of NAFLD. Studies have demonstrated that corn peptides exert hepatoprotective effects in NAFLD by mitigating oxidative stress and lipid accumulation through activation of the AMPKα/SIRT1, SIRT1/PPAR-α, and Nrf2/HO-1 pathways [18,19]. These findings underscore the potential of plant proteins as functional ingredients and therapeutic strategies for improving lipid homeostasis and combating NAFLD.

Pea albumin (PA) is a water-soluble protein extracted from peas, gaining attention for its excellent nutritional composition and functional properties. Initially, research on PA primarily focused on its physicochemical characteristics and processing properties [20,21,22]. In recent years, PA has drawn significant interest due to its richness in essential amino acids and branched-chain amino acids, giving it notable potential in preventing and treating hepatic lipid accumulation and alleviating obesity. Studies have shown that PA can influence lipid metabolism by modulating critical pathways associated with lipid synthesis and degradation [23]. Previous reports have shown that PA can inhibit lipid accumulation and restore gut microbiota balance, thereby preventing obesity and alleviating metabolic disorders in mice induced by a high-fat diet [24]. An in vitro study also reported that PA hydrolysates markedly reduce lipid accumulation in 3T3-L1 cells [25]. Furthermore, PA possesses antioxidant and anti-inflammatory properties, which may further contribute to its potential role in metabolic regulation. PA exhibits antioxidant activity by scavenging free radicals and reducing oxidative stress-induced damage to cells [26]. In a study, PA was found to downregulate NF-κB and STAT3 signaling pathways and restore gut microbiota diversity, thereby alleviating DSS-induced colitis [27]. The research studies indicate that PA exhibits potential activity in reducing hepatic lipid accumulation, while its mechanisms for regulating hepatic lipid metabolism remain unclear. Given the rising prevalence of NAFLD and the limitations of current treatments, PA represents a viable alternative and warrants further investigation as a functional ingredient for the prevention and management of lipid metabolism disorders.

This study aims to investigate the inhibitory effects of PA on lipid accumulation in LO2 cells induced by oleic acid (OA). By exploring the effects of PA on key regulatory proteins involved in lipid synthesis, breakdown, and oxidation, we aim to elucidate the mechanisms through which PA regulates lipid metabolism and reduces lipid accumulation. This research provides new insights into the potential application of PA as a functional ingredient for the prevention or treatment of NAFLD.

## 2. Materials and Methods

### 2.1. Materials and Reagents

Pea seeds (*Pisum sativum* L.) were provided by Yantai Shuangta Food Co., Ltd. (Yantai, China). OA was purchased from Sigma-Aldrich (St. Louis, MO, USA). Atorvastatin (ATO) was purchased from Pfizer Pharmaceuticals Limited (New York, NY, USA). Bovine serum albumin (BSA) was acquired from Solarbio Technology Co., Ltd. (Beijing, China). TG kit, total cholesterol (TC) kit, aspartate aminotransferase (AST) kit, alanine aminotransferase (ALT) kit, and superoxide dismutase (SOD) kit were purchased from Nanjing Jiancheng Bioengineering Institute (Nanjing, China). Cell counting kit-8 (CCK-8), modified Oil Red O Staining kit, glutathione peroxidase (GSH-Px) kit, and malondialdehyde (MDA) kit were purchased from Beyotime (Shanghai, China). The primary rabbit antibodies against AMPKα, p-AMPKα, SREBF1, ACC, p-ACC, FASN, PPARα, PPARγ, GAPDH, and β-Actin were obtained from Beyotime (Shanghai, China). The primary rabbit antibodies against ATGL, HSL, MGLL, ACSL, CPT-1, and CPT-2 were obtained from Proteintech (Chicago, IL, USA). DMEM medium, penicillin–streptomycin, fetal bovine serum (FBS), and 0.25% trypsin-EDTA were purchased from Gibco Company (New York, NY, USA). All other chemical reagents and solvents used in the experiments were of an analytical reagent grade.

### 2.2. Preparation of Pea Albumin (PA)

A prior study outlined a method for extracting PA from pea seeds [23]. Pea seeds were first crushed, defatted, and then subjected to alkali dissolution and acid precipitation to extract protein. After dialysis and salt precipitation, PA powder was obtained by freeze-drying. The moisture content of the extracted PA was 3.87%, the dry matter and protein content values were 96.13% and 92.65%, and the starch, ash, and total dietary fiber content values were 0.14%, 2.78%, and 0.26%. PA contains approximately 16.71% essential amino acids, 25.20% glycine, and 7.34% branched-chain amino acids.

### 2.3. Cell Culture

LO2 cells were purchased from the American Type Culture Collection. LO2 cells, originally derived from human liver cells, were later reported to be contaminated with HeLa cells [28]. Despite this, LO2 cells have remained a commonly used model in studies related to lipid metabolism [19,29]. LO2 cells were cultured in DMEM medium supplemented with 10% FBS and 1% penicillin–streptomycin. The cells were incubated at 37 °C in a 5% CO_2_ atmosphere.

### 2.4. Cell Viability Assay

Cell viability of LO2 cells was evaluated using the CCK-8 assay. Cells were seeded in 96-well plates at a density of 1 × 10^4^ cells per well and incubated for 24 h. Subsequently, the cells were treated with varying doses of OA (0, 200, 300, 400, 500, 600, 700, 800, 900, 1000 μM), PA (0, 100, 200, 400, 600, 800, 1000, 1200, 1400, 1600 μg/mL) or ATO (0, 1, 2, 4, 6, 8, 10, 20 μM) for 24 h, followed by a 60 min incubation at 37 °C with 10 µL of CCK-8 solution. Absorbance was measured at 450 nm using a microplate reader (BioTek Ltd., Winooski, VT, USA). Cell viability was expressed as a percentage, comparing the absorbance of treated cells to that of untreated cells. Based on the results of cell viability, the modeling concentration of OA and the safe concentrations of PA and ATO for the intervention in LO2 cells were determined.

### 2.5. Cell Treatment

The lipid accumulation model in LO2 cells was established using OA, following a previously reported method [30]. ATO was included as a positive control, as previous studies have demonstrated its efficacy in alleviating lipid accumulation [31,32], thereby serving as a benchmark for evaluating the effects of our interventions. Cell viability assays were employed to identify the optimal concentration of OA for model induction, as well as the appropriate intervention concentrations of PA and ATO. Cells were divided into six groups for intervention: (1) normal control (NC) group, cultured in DMEM medium; (2) model control (OA) group, cultured in DMEM medium containing 500 μM OA; (3) low-dose PA (PA-L) group, cultured in DMEM medium with 500 μM OA and supplemented with 100 μg/mL PA; (4) medium-dose PA (PA-M) group, cultured in DMEM medium with 500 μM OA and supplemented with 300 μg/mL PA; (5) high-dose PA (PA-H) group, cultured in DMEM medium with 500 μM OA and supplemented with 600 μg/mL PA; (6) positive control (ATO) group, cultured in DMEM medium with 500 μM OA and supplemented with 4 μM ATO. After 24 h of incubation, cells and culture supernatants were harvested for subsequent analysis.

### 2.6. Oil Red O Staining

The cell staining procedure was conducted following the manufacturer’s instructions (provided with the Oil Red O Staining kit). After 24 h of intervention in LO2 cells, the cells were washed twice with 1 mL of PBS. Cell fixative was added to the well plates and left to stand for 20 min to fix the cells. After cell fixation, the fixative was discarded and the cells were covered with 500 μL of staining solution per well for 30 min. The dye solution was discarded and 1 mL of distilled water was added to each well to remove any remaining dye solution. The cells were re-stained with hematoxylin for 1–2 min and then washed with distilled water 2–5 times until there was no residual dye solution. After rinsing, 1 mL of distilled water was added to each well, and observations were made via a microscope (Leica Microsystems, Wetzlar, Germany).

### 2.7. Determination of Cellular Lipid Indicators

LO2 cells were inoculated in 12-well plates and cultured until the cell density reached 60%, then the intervention culture was performed, and the cell precipitate was collected after 24 h. The cells were resuspended in PBS buffer and centrifuged at 200× *g* for 10 min, after which the supernatant was discarded. PBS buffer was added to the cell precipitate and broken by ultrasonication under ice bath conditions. The absorbance at 500 nm was detected according to the instructions of TG and TC assay kits, while protein concentration was assessed using the BCA kit for normalization calculations. The TG and TC contents in LO2 cells were finally determined.

### 2.8. Determination of Cell Damage Indicators

After treatments, the cell supernatants were taken and the absorbance was measured at 510 nm according to the instructions of the ALT and AST kits. Protein concentration was also determined using the BCA kit for normalization calculations.

### 2.9. Measurement of Cellular Oxidative Stress Indicators

After treatments, the precipitate was collected and resuspended with PBS buffer. Following centrifugation at 200× *g* for 10 min, the supernatant was discarded. PBS buffer was added to the cell sediment and broken by sonication under ice bath conditions. Subsequently, the absorbance was measured according to the instructions of MDA, SOD, and GSH-Px kits, while protein concentration was assessed using the BCA kit (Solarbio, Beijing, China) for normalization calculations.

### 2.10. Western Blot Analysis

After treatment, the cell precipitate with the medium removed was collected and an appropriate amount of cell lysate containing a mixture of protein phosphatase inhibitors was added. The cells were lysed on ice for 20 min, then sonicated in an ice bath, followed by centrifugation at 4 °C for 15 min at 12,000× *g*. The supernatant was collected as the cellular proteins. Equal amounts of protein samples were separated on 10% SDS-PAGE gels and subsequently transferred to PVDF membranes. The membranes were blocked with 5% BSA and incubated overnight at 4 °C with specific primary antibodies (p-AMPKα, AMPKα, p-ACC, ACC, SREBF1, FASN, PPARγ, ATGL, HSL, MGLL, PPARα, ACSL, CPT-1, CPT-2, GAPDH, and β-Actin). After washing, the membranes were incubated with secondary antibodies at room temperature for 1 h. Following this incubation, the PVDF membranes were washed again. Protein bands were then visualized using an ECL kit. Protein expression levels were quantified using ImageJ version 1.8.0 software, normalizing against GAPDH and β-Actin.

### 2.11. Statistical Analysis

Analysis was performed using SPSS 23.0 software. The results are expressed as mean ± SD. Data normality was assessed using the Shapiro–Wilk test. For normally distributed data, one-way analysis of variance (ANOVA) was employed, followed by the LSD post hoc test to determine statistical differences between groups. For data that did not follow a normal distribution, the Kruskal–Wallis test was applied. *p* < 0.05 was considered statistically significant. Graphs were generated using GraphPad Prism 9.2.0.

## 3. Results

### 3.1. OA, PA, and ATO Effects on the Viability of LO2 Cells

**Figure 1:**
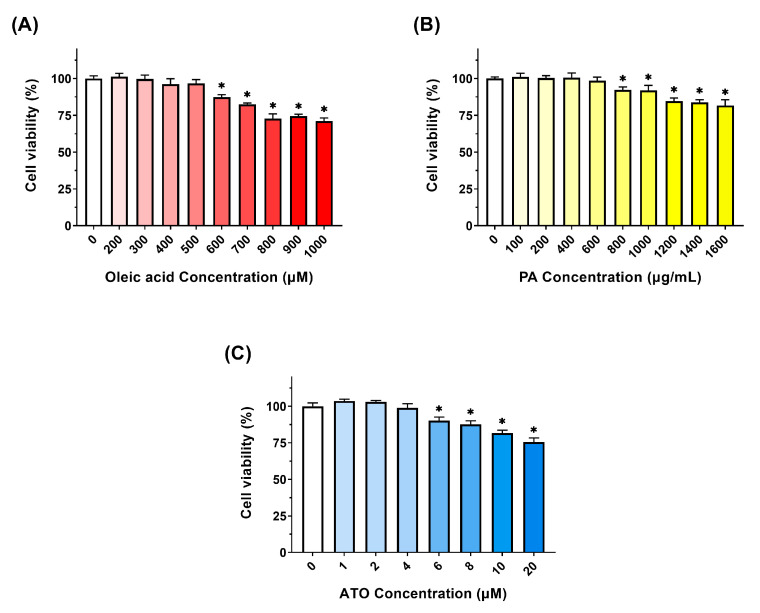
Effects of different concentrations of OA (**A**), PA (**B**), and ATO (**C**) on the viability of LO2 cells. Results are expressed as mean ± SD. *n* = 5 per group. * *p* < 0.05, there is a significant difference between the intervention groups with different concentrations and the control group. (OA: oleic acid; PA: pea albumin; ATO: atorvastatin).

This study treated LO2 cells with different concentrations of OA, PA, and ATO for 24 h and measured cell viability to determine the modeling concentration of OA as well as the safe concentrations of PA and ATO for the intervention in LO2 cells. As shown in Figure 1A, no significant inhibition of cell proliferation was observed when the OA concentration was less than 500 μM, whereas the cell viability decreased significantly when the OA concentration was greater than 500 μM (*p* < 0.05) and the inhibitory effect increased with the increase of OA concentration. When the PA concentration exceeded 600 μg/mL, there was a significant decrease in LO2 cell viability (*p* < 0.05), which occurred in a dose-dependent manner (Figure 1B). ATO was used as a positive control, with LO2 cell viability significantly declining at concentrations above 4 μM (*p* < 0.05). And as the concentration of ATO increases, its inhibitory effect on cell proliferation becomes increasingly significant (Figure 1C). Therefore, in this study, the final modeling concentration of OA was set at 500 μM, and the maximum intervention concentration of PA and ATO was set at 600 μg/mL and 4 μM. The intervention doses of PA in the follow-up experiments were 100, 300, and 600 μg/mL.

### 3.2. Effects of PA on OA-Induced Lipid Accumulation in LO2 Cells

**Figure 2:**
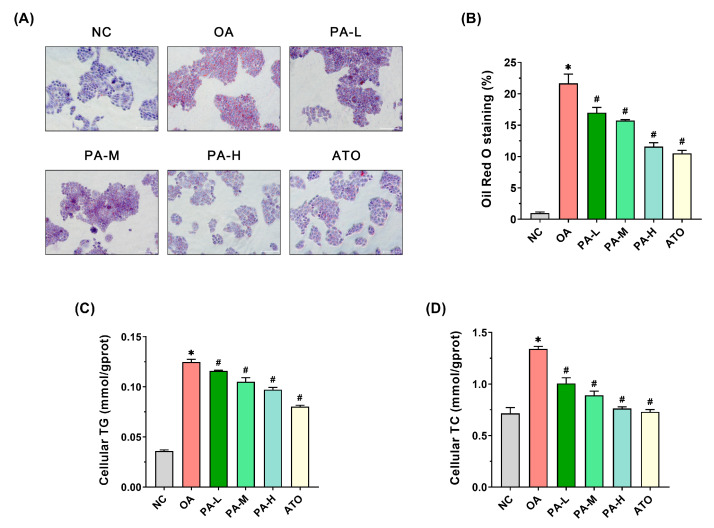
Effects of PA on OA-induced lipid accumulation in LO2 cells. (**A**) Oil Red O staining of LO2 cells (Scale bar = 200 μm). (**B**) Positive area of Oil Red O staining in LO2 cells. (**C**) TG content in LO2 cells. (**D**) TC content in LO2 cells. Results are expressed as mean ± SD. *n* = 3 per group. * *p* < 0.05, OA vs. NC; ^#^ *p* < 0.05, OA vs. PA-L, PA-M, PA-H, or ATO. (NC: normal control group; OA: model control group; PA-L: low-dose pea albumin group; PA-M: medium-dose pea albumin group; PA-H: high-dose pea albumin group; ATO: positive control group; TG: triglyceride; TC: total cholesterol).

Oil Red O staining, a standard technique for assessing cellular lipid accumulation, was employed in this study to evaluate the impact of PA on OA-induced lipid accumulation in LO2 cells. As shown in Figure 2A, after staining, the NC group showed almost no red lipid droplets, while the OA group exhibited significant lipid droplet aggregation around the cells, confirming the successful induction of the lipid accumulation model in LO2 cells by OA. In the PA-L, PA-M, and PA-H groups, the number of red lipid droplets progressively decreased, with a marked reduction in lipid droplet aggregation. Similarly, the ATO group showed a significant decline in pericellular lipid droplets. The quantitative analysis of Oil Red O staining indicated that the OA group exhibited a significantly greater area of positive staining compared to the NC group (Figure 2B, *p* < 0.05). The PA intervention led to a dose-dependent decrease in positive staining areas in the PA-L, PA-M, and PA-H groups (*p* < 0.05), with high-dose PA and ATO showing comparable effects.

In addition, this study also assessed the TG and TC levels in LO2 cells subjected to various treatments. The results of TG content are shown in Figure 2C. The TG content in the cells of the OA group was about 3.47 times higher than that of the NC group. In comparison to the OA group, the TG content in the cells of the PA-L, PA-M, and PA-H groups decreased in a dose-dependent manner, while the TG levels in the ATO group also showed a significant reduction (*p* < 0.05). Among them, the high-dose PA intervention and ATO intervention significantly reduced 22.11% and 35.76%, respectively, compared to the OA group, but the intracellular TG content of the two intervention groups still did not reach the normal cellular level. The results of TC content were shown in Figure 2D, which was significantly increased by about 1.87-fold in the cells of the OA group compared to the NC group. Compared to the OA group, the TC content in the cells of the PA-L, PA-M, and PA-H groups decreased in a dose-dependent manner, in which the high-dose of PA showed the best effect among the three dose groups, with a reduction in TC content of about 43.18% (*p* < 0.05). The cellular TC content was significantly reduced by 45.55% after ATO intervention (*p* < 0.05). And the cellular TC content had converged to normal cells after high-dose PA and ATO intervention.

### 3.3. Effects of PA on OA-Induced Cell Damage in LO2 Cells

**Figure 3:**
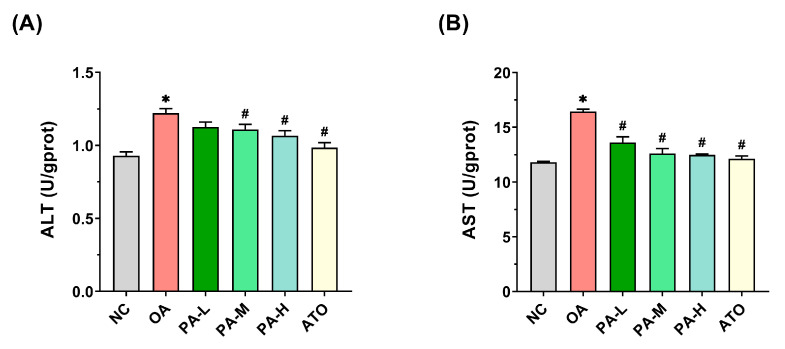
Effects of PA on OA-induced cell damage in LO2 cells. (**A**) ALT activity in the culture supernatant of LO2 cells. (**B**) AST activity in the culture supernatant of LO2 cells. Results are expressed as mean ± SD. *n* = 3 per group. * *p* < 0.05, OA vs. NC; ^#^ *p* < 0.05, OA vs. PA-L, PA-M, PA-H, or ATO. (NC: normal control group; OA: model control group; PA-L: low-dose pea albumin group; PA-M: medium-dose pea albumin group; PA-H: high-dose pea albumin group; ATO: positive control group; ALT: alanine aminotransferase; AST: aspartate aminotransferase).

ALT and AST activities serve as key biomarkers for liver injury. To assess whether OA induces damage in LO2 cells and evaluate the protective effects of PA intervention, this study measured the levels of ALT and AST in the cell culture supernatants. As illustrated in Figure 3A, OA treatment resulted in a significant increase in ALT activity in the cell culture supernatants compared to the NC group (*p* < 0.05), with ALT levels rising to 1.39 times those of the NC group. While ALT activity in the PA-L group did not show a notable reduction compared to the OA group (*p* > 0.05), significant decreases were observed in the PA-M, PA-H, and ATO groups (*p* < 0.05). Figure 3B demonstrates that AST activity in the OA group was significantly elevated (*p* < 0.05), reaching 1.31 times that of the NC group. In contrast to the OA group, AST levels in the PA-L, PA-M, PA-H, and ATO groups were significantly reduced (*p* < 0.05), with AST activity in the PA-H and ATO groups approaching that of the NC group. These findings suggest that PA intervention effectively lowered ALT and AST activities in LO2 cell culture supernatants, thereby mitigating OA-induced LO2 cell damage.

### 3.4. Effects of PA on OA-Induced Oxidative Stress in LO2 Cells

**Figure 4:**
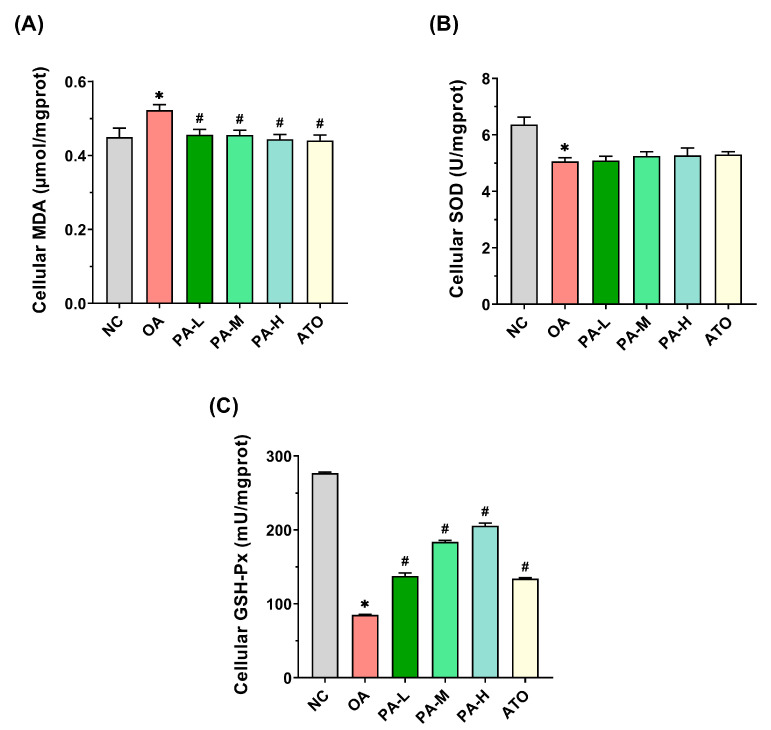
Effects of PA on OA-induced oxidative stress in LO2 cells. (**A**) MDA content in LO2 cells. (**B**) SOD activity in LO2 cells. (**C**) GSH-Px activity in LO2 cells. Results are expressed as mean ± SD. *n* = 3 per group. * *p* < 0.05, OA vs. NC; ^#^ *p* < 0.05, OA vs. PA-L, PA-M, PA-H, or ATO. (NC: normal control group; OA: model control group; PA-L: low-dose pea albumin group; PA-M: medium-dose pea albumin group; PA-H: high-dose pea albumin group; ATO: positive control group; MDA: malondialdehyde; SOD: superoxide dismutase; GSH-Px: glutathione peroxidase).

To assess the effects of PA on OA-induced oxidative stress in LO2 cells, the levels of MDA, along with the activities of SOD and GSH-Px were measured. As illustrated in Figure 4A, OA treatment significantly elevated MDA levels in LO2 cells (*p* < 0.05), with the OA group exhibiting MDA content approximately 1.16 times higher than that of the NC group. In contrast, MDA levels were significantly reduced in the PA-L, PA-M, PA-H, and ATO groups compared to the OA group (*p* < 0.05), with decreases of 15.11% and 15.65% observed in the PA-H and ATO groups, respectively. As demonstrated in Figure 4B, SOD activity in the OA group decreased significantly by 20.53% compared to the NC group (*p* < 0.05). Although SOD activity increased following PA and ATO interventions, the differences were not statistically significant (*p* > 0.05). As shown in Figure 4C, GSH-Px activity in the OA group was significantly reduced by approximately 69.27% compared to the NC group (*p* < 0.05). However, GSH-Px activity was significantly elevated in the PA-L, PA-M, PA-H, and ATO groups compared to the OA group (*p* < 0.05). Notably, PA intervention increased GSH-Px activity in a dose-dependent manner, with the highest levels observed in the PA-H group, where GSH-Px activity was 2.42 times higher than that of the OA group, surpassing the effect of ATO intervention. The results demonstrated that PA intervention significantly decreased MDA levels in OA-induced LO2 cells while enhancing GSH-Px activity. This suggests that PA effectively mitigates oxidative stress in LO2 cells induced by OA.

### 3.5. PA Inhibits OA-Induced Lipid Accumulation in LO2 Cells by Regulating Lipid Synthesis Pathways

**Figure 5:**
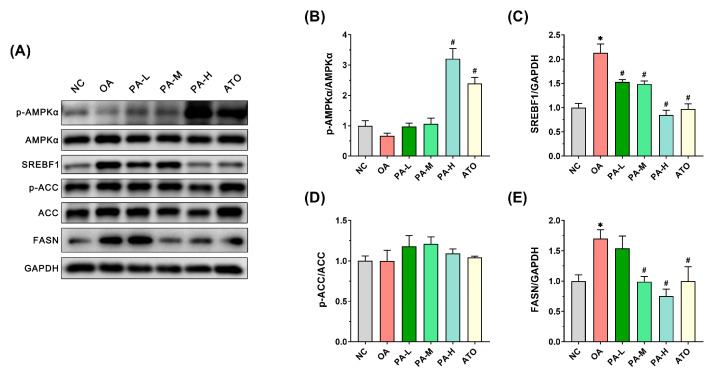
Effects of PA on the expression of lipid synthesis-related proteins in OA-induced LO2 cells. (**A**) Representative Western blots of markers for lipogenesis in LO2 cells. (**B**–**E**) Quantification of protein expression of *p*-AMPKα/AMPKα, SREBF1, *p*-ACC/ACC, and FASN. Results are expressed as mean ± SD. *n* = 3 per group. * *p* < 0.05, OA vs. NC; ^#^ *p* < 0.05, OA vs. PA-L, PA-M, PA-H, or ATO. (NC: normal control group; OA: model control group; PA-L: low-dose pea albumin group; PA-M: medium-dose pea albumin group; PA-H: high-dose pea albumin group; ATO: positive control group).

To better elucidate the mechanism through which PA inhibits lipid accumulation in LO2 cells, we analyzed the expression levels of key proteins involved in the lipid synthesis pathway, as shown in Figure 5A. As indicated in Figure 5B, the phosphorylation level of AMPKα protein did not differ significantly between the OA group and the NC group (*p* > 0.05). However, following high-dose PA and ATO intervention, the phosphorylation of AMPKα was significantly elevated, with increases of 4.82-fold and 3.59-fold, respectively, compared to the OA group (*p* < 0.05). The relative expression levels of the regulatory factor SREBF1 protein are shown in Figure 5C. In the OA group, SREBF1 expression was significantly elevated (*p* < 0.05), at 2.13 times that of the NC group. Compared to the OA group, SREBF1 expression values were reduced by 28.17%, 30.23%, and 60.23% in the PA-L, PA-M, and PA-H groups (*p* < 0.05), respectively. Additionally, the ATO group showed a 54.23% reduction in SREBF1 expression (*p* < 0.05). As shown in Figure 5D, no significant differences were observed in the phosphorylation levels of ACC protein between the groups (*p* > 0.05). However, PA intervention led to an increase in the phosphorylation of ACC protein. FASN is a key protein involved in lipid synthesis in LO2 cells, and its relative expression is shown in Figure 5E. In the OA group, FASN expression was significantly elevated (*p* < 0.05), reaching 1.7 times that of the NC group. Compared to the OA group, the expression levels of the FASN protein in the PA-M and PA-H groups decreased by 41.76% and 55.62% (*p* < 0.05), respectively. Additionally, the ATO group exhibited a 41.17% reduction in FASN expression (*p* < 0.05) compared to the OA group. These findings suggest that PA inhibits lipid synthesis in LO2 cells by modulating AMPKα phosphorylation and downregulating the expression of SREBF1 and FASN proteins.

### 3.6. PA Inhibits OA-Induced Lipid Accumulation in LO2 Cells by Regulating TG Catabolism Pathways

**Figure 6:**
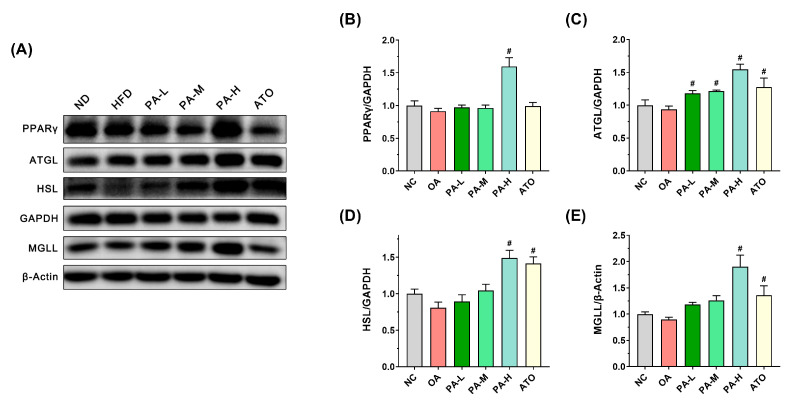
Effects of PA on the expression of TG catabolism-related proteins in OA-induced LO2 cells. (**A**) Representative Western blots of markers for TG catabolism in LO2 cells. (**B**–**E**) Quantification of protein expression of PPARγ, ATGL, HSL, and MGLL. Results are expressed as mean ± SD. *n* = 3 per group. ^#^ *p* < 0.05, OA vs. PA-L, PA-M, PA-H, or ATO. (NC: normal control group; OA: model control group; PA-L: low-dose pea albumin group; PA-M: medium-dose pea albumin group; PA-H: high-dose pea albumin group; ATO: positive control group).

TG catabolism is essential for mitigating lipid accumulation in LO2 cells, with PPARγ acting as a key regulator of hepatocyte lipolysis. The process involves three major rate-limiting enzymes: ATGL, HSL, and MGLL [33]. To explore the impact of PA on TG catabolism, we assessed the expression levels of key proteins in the lipolysis pathway in LO2 cells, as shown in Figure 6. The results indicated that the relative expression levels of TG catabolism-related proteins, PPARγ, ATGL, HSL, and MGLL were not significantly different between the OA and NC groups (Figure 6B–E, *p* > 0.05). However, compared to the OA group, following high-dose PA intervention, the expression levels of PPARγ, ATGL, HSL, and MGLL significantly increased by 1.75-fold, 1.43-fold, 1.85-fold, and 2.12-fold (*p* < 0.05), respectively. In contrast, ATO intervention did not significantly affect PPARγ expression compared to the OA group (*p* > 0.05), but it significantly elevated the expressions of ATGL, HSL, and MGLL (*p* < 0.05). These results indicate that high-dose PA intervention effectively promoted the expression of TG catabolism-related proteins in LO2 cells, demonstrating a superior effect compared to ATO intervention.

### 3.7. PA Inhibits OA-Induced Lipid Accumulation in LO2 Cells by Regulating Fatty Acid Oxidation Pathways

**Figure 7:**
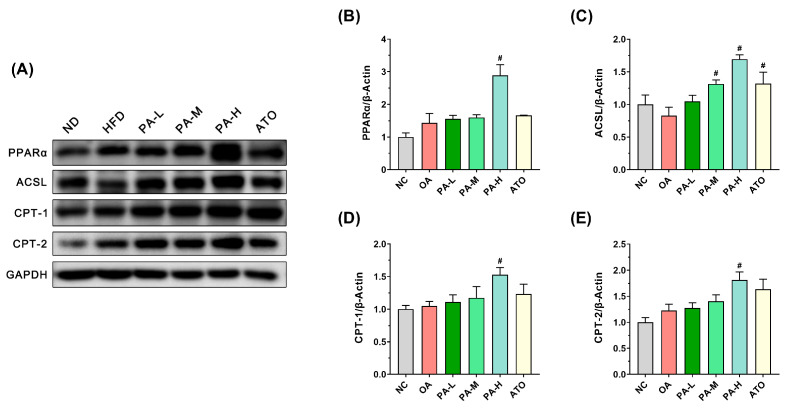
Effects of PA on the expression of fatty acid oxidation-related proteins in OA-induced LO2 cells. (**A**) Representative Western blots of markers for fatty acid oxidation in LO2 cells. (**B**–**E**) Quantification of protein expression of PPARα, ACSL, CPT-1, and CPT-2. Results are expressed as mean ± SD. *n* = 3 per group. ^#^ *p* < 0.05, OA vs. PA-L, PA-M, PA-H, or ATO. (NC: normal control group; OA: model control group; PA-L: low-dose pea albumin group; PA-M: medium-dose pea albumin group; PA-H: high-dose pea albumin group; ATO: positive control group).

Fatty acid β-oxidation in mitochondria is a crucial pathway for lipid metabolism in LO2 cells. To assess the impact of PA on fatty acid oxidation capacity, the expression levels of key proteins involved in this process, PPARα, ACSL, CPT-1, and CPT-2 were evaluated in LO2 cells, as depicted in Figure 7. As shown in Figure 7B-E, the relative expressions of fatty acid oxidation-related proteins PPARα, ACSL, CPT-1, and CPT-2 in the OA group were not significantly different from those in the NC group (*p* > 0.05). Compared to the OA group, PA intervention resulted in a dose-dependent increase in the relative expressions of PPARα, ACSL, CPT-1, and CPT-2 proteins. Notably, high-dose PA intervention significantly enhanced the expression of these proteins (*p* < 0.05), with increases of 2.02-fold, 2.05-fold, 1.46-fold, and 1.48-fold, respectively, compared to the OA group. Following ATO intervention, there was no significant change in the relative expression of PPARα protein (*p* > 0.05). While the relative expressions of CPT-1 and CPT-2 proteins showed a tendency to increase, the differences were not significant (*p* > 0.05). However, the relative expression of ACSL protein increased significantly compared to the OA group (*p* < 0.05). These results indicate that PA intervention enhanced the expression of proteins involved in fatty acid β-oxidation in LO2 cells, thereby promoting β-oxidation and reducing lipid accumulation. Notably, high-dose PA demonstrated a superior effect compared to ATO.

## 4. Discussion

This study investigates the mechanism by which PA inhibits lipid accumulation and regulates lipid metabolism in LO2 cells using an OA-induced lipid accumulation model. The modeling concentration of 500 μM OA successfully induced lipid accumulation in LO2 cells, as evidenced by the significant increase in intracellular TG and TC content, and lipid droplet aggregation, as observed in Oil Red O staining. This finding aligns with previous studies that employed OA to model steatosis in hepatocytes [34]. In this study, PA intervention, particularly at high doses, significantly reduced lipid droplet formation, TG, and TC levels, with effects comparable to the positive control, ATO. Liu et al. found that PA could inhibit lipid accumulation in 3T3-L1 cells by decreasing TG content during cell differentiation [24]. Ruiz et al. also studied and compared the effects of two hydrolysates of PA and whey protein on the accumulation of lipids in cells, which confirms our idea [25]. These findings suggest that PA may effectively inhibit OA-induced lipid accumulation through a mechanism similar to that of pharmacological agents used to treat dyslipidemia. Prolonged elevation of intracellular lipid content disrupts lipid metabolism, leading to excessive lipid droplet accumulation and lipid peroxidation, which ultimately causes cellular damage. Elevated levels of ALT and AST are indicative of hepatocyte damage. In this study, PA intervention significantly reduced ALT and AST activity in the cell culture supernatant compared to the OA group, suggesting that PA effectively alleviates OA-induced LO2 cell damage. Lipid accumulation induces lipotoxicity, leading to subsequent oxidative stress. This process results in elevated levels of the toxic metabolite MDA and a decrease in the activity of antioxidant enzymes such as SOD and GSH-Px, ultimately causing mitochondrial dysfunction [35]. Since mitochondria are crucial for fatty acid β-oxidation in hepatocytes, their impairment further hinders lipid oxidative metabolism and exacerbates lipid accumulation [36]. OA treatment notably increased MDA levels while decreasing SOD and GSH-Px activity, indicating elevated oxidative stress in LO2 cells. In this study, PA was found to lower MDA levels and enhance GSH-Px activity, thus alleviating OA-induced oxidative stress in LO2 cells. This finding aligns with the reports that pea protein hydrolysate enhances antioxidant activity in HepG2 cells [37]. Notably, high-dose PA exhibited superior effects compared to ATO, suggesting that PA may provide enhanced protective benefits against oxidative stress in the LO2 cells. Taken together, these findings suggest that PA could ameliorate OA-induced lipid accumulation in LO2 cells.

To investigate the inhibitory mechanism of PA on lipid accumulation in LO2 cells, the expression of key proteins of the lipid metabolic pathway in LO2 cells was analyzed. Further analysis of the lipid synthesis pathway revealed that PA significantly increased the phosphorylation of AMPKα, a critical regulator of energy homeostasis and lipid metabolism. AMPKα activation suppresses lipid synthesis by inhibiting SREBF1 and FASN expression, both of which were significantly downregulated following PA intervention. The suppression of FASN, a key enzyme involved in fatty acid synthesis, likely contributed to the reduced lipid accumulation observed in LO2 cells. These results are in agreement with previous studies that have highlighted the role of AMPK in regulating hepatic lipid metabolism [38]. In addition to inhibiting lipid synthesis, PA also promoted TG catabolism by upregulating the expression of key proteins involved in lipolysis, including PPARγ, ATGL, HSL, and MGLL. PPARγ plays a crucial role in lipid catabolism, and its upregulation likely contributed to the enhanced lipolysis observed in this study [39]. The activation of ATGL, HSL, and MGLL in hepatocytes can promote the breakdown of TG in the citric acid cycle, forming glycerol and free fatty acids to generate energy, playing a crucial role in regulating hepatic lipid accumulation [40,41]. Notably, the high-dose PA intervention significantly enhanced the expression of these proteins, surpassing the effects of ATO. These results suggest that PA may serve as a potent activator of lipolysis, providing a dual benefit in both reducing lipid synthesis and promoting lipid breakdown. Furthermore, the role of PA in enhancing fatty acid oxidation was evident from the upregulation of PPARα, ACSL, CPT-1, and CPT-2 expression. These proteins are crucial for mitochondrial fatty acid β-oxidation, a process that contributes to lipid clearance in hepatocytes. PPARα regulates the expression of genes involved in fatty acid oxidation, enhancing the breakdown of fatty acids into acetyl-CoA [42]. ACSL initiates fatty acid oxidation by converting free fatty acids into acyl-CoA [43], while CPT-1 and CPT-2 facilitate the mitochondrial transport and conversion of acylcarnitines, respectively [44,45]. Together, these proteins orchestrate efficient fatty acid metabolism, which is crucial for modulating lipid accumulation in hepatic cells. PA significantly increased the expression of these proteins, particularly at high doses, further supporting its role in reducing lipid accumulation in LO2 cells. Additionally, fatty acid binding proteins (FABPs) and CD36 play significant roles in fatty acid transport and metabolism. FABPs are essential for the intracellular binding and transport of fatty acids, while CD36 facilitates the uptake of long-chain fatty acids into cells [46,47]. However, our research did not investigate the expression levels of FABP and CD36. Due to their potential importance, we will explore the regulatory effects of PA on these proteins in future studies. Liu et al. also demonstrated that PA could influence lipid metabolism by modulating the expression of proteins associated with lipolysis and fatty acid oxidation in adipose tissue [24]. Interestingly, ATO did not significantly enhance PPARα expression, underscoring the unique ability of PA to regulate this pathway. Our results demonstrated that high-dose PA intervention was able to regulate lipid metabolism in LO2 cells by modulating the expression of key proteins in lipid synthesis, TG catabolism, and fatty acid oxidation in LO2 cells, which led to the improvement of OA-induced lipid accumulation in LO2 cells. NAFLD is characterized by the accumulation of fats, particularly TG, in hepatocytes. Studies show that liver lipid metabolism imbalances are more pronounced under protein deficiency. Ampong et al. discovered that a low-protein diet is associated with excessive storage of triglycerides and the development of NAFLD [48]. Although this study was conducted under normal protein conditions, PA’s regulatory effects suggest broader applications, especially in protein-deficient scenarios. Future research should explore these effects in animal models or clinical trials under different dietary conditions, providing stronger support for developing PA-based functional foods to improve lipid metabolism and alleviate NAFLD.

## 5. Conclusions

Taken together, PA was able to ameliorate OA-induced lipid accumulation in LO2 cells by reducing intracellular lipid droplet aggregation and lowering cellular TG and TC content. It also ameliorated OA-induced cellular damage and oxidative stress response in LO2 cells, reduced cellular ALT and AST secretion, decreased cellular MDA content, and increased GSH-Px viability. PA’s ability to inhibit lipid synthesis, enhance lipolysis, and promote fatty acid β-oxidation suggests its potential as a functional food ingredient or dietary supplement component for managing hepatic steatosis. While these findings provide a mechanistic understanding of PA’s effect on lipid metabolism in cells, future studies will investigate its role in dietary interventions within more complex nutritional environments, including potential interactions with other nutrients.

## Data Availability

The original contributions presented in this study are included in the article. Further inquiries can be directed to the corresponding author.
